# Characterization and phylogenetic analysis of the complete mitochondrial genome of a tropical sea cucumber, *Holothuria fuscocinerea*

**DOI:** 10.1080/23802359.2020.1787264

**Published:** 2020-07-06

**Authors:** Chuhang Cheng, Feifei Wu, Chunhua Ren, Xiao Jiang, Xiaofen Wu, Wen Huang, Chaoqun Hu

**Affiliations:** a CAS Key Laboratory of Tropical Marine Bio-resources and Ecology (LMB)/Guangdong Provincial Key Laboratory of Applied Marine Biology (LAMB), South China Sea Institute of Oceanology, Chinese Academy of Sciences, Guangzhou, China; b University of Chinese Academy of Sciences, Beijing, China; c Laboratory of Aquatic Sciences, Key Laboratory of Animal Nutrition and Feed Science in South China of Ministry of Agriculture and Rural Affairs, Guangdong Key Laboratory of Animal Breeding and Nutrition, Institute of Animal Science, Guangdong Academy of Agricultural Sciences, Guangzhou, China; d Chinese Academy of Sciences, Institution of South China Sea Ecology and Environmental Engineering (ISEE), Guangzhou, China

**Keywords:** Mitochondrial genome, Holothuria fuscocinerea, Phylogenetic analysis, 16S rRNA

## Abstract

In this study, the mitochondrial genome (mitogenome) of *Holothuria fuscocinerea* was unraveled to be 15,890 bp in length, containing 13 protein-coding genes (PCGs), 22 tRNA genes, and 2 rRNA genes. The PCGs were initiated by four initiation codons (ATG, TAC, ATC, and ATA). Only one PCG (*nad6*) and five tRNA genes (*tRNA^Ser(UCN)^*, *tRNA^Gln^*, *tRNA^Ala^*, *tRNA^Val^,* and *tRNA^Asp^*) were encoded on the light chain, and the other genes were encoded on the heavy chain. A phylogenetic tree constructed with 16S rRNA sequences showed that *H. fuscocinerea* is most closely related to *H. leucospilota*.

*Holothuria fuscocinerea* (Echinodermata: Holothuroidea, *H. fuscocinerea*) is naturally distributed near boulders, corals, and seaweed clumps in the West Pacific, East Africa, Australia, and Southeastern China (Liao 1997), and it might play important roles in maintaining a healthy coral reef ecosystem (Birkeland 1989; Schneider et al. 2011).

Compared with whole mitochondrial genes, a fragment of a single mitochondrial gene such as 16S rRNA can provide more information in terms of identification and evolution (Kerr et al. 2005; Byrne et al. 2010; Liu et al. 2016; Zou et al. 2017; Liu et al. 2018). The classification of sea cucumber from the mitochondrial genome (mitogenome) level would be more accurate than the aspect of phenotypic characteristics such as the morphology of the tentacles, the endoskeleton, and the calcareous ring (Liao 1997; Rowe and Richmond 2004).

*Holothuria fuscocinerea* was obtained from Daya Bay (N22°35′, E114°31′), Shenzhen, Guangdong, China. The specimen was stored in the Marine Biotechnology and Disease Control Laboratory of the South China Sea at the Chinese Academy of Sciences in Guangzhou, China (MBDC170711124). Total DNA was extracted and sent to BGI Genomics Co., Ltd., China, for sequencing. After aligning, splicing, correction, and identification of protein-coding genes and tRNA genes, the obtained mitogenome DNA sequence was analyzed by phylogenetic analysis.

The mitogenome of *H. fuscocinerea* (MK391177) show a double-strand molecule of 15,890 bp (33.1% A, 27.3% T, 23.9% C, and 15.7% G), including a set of 22 tRNA genes that varied from 61 bp (*tRNA^Lys^*) to 72 bp (*tRNA^Leu(UUR)^*) in length (the total length of tRNAs was 1,511 bp), 13 protein-coding genes that consisted of 3,690 codons, 2 rRNAs (*lrRNA*: GC% = 38.07%, *srRNA*: GC% = 43.48%), and a putative noncoding control region between *tRNA^Thr^* and *tRNA^Pro^*(GC% = 59.01%).

The phylogenetic analyzing demonstrated that *H. fuscocinerea* most closely related to *H. leucospilota* (Figure 1). The phylogenetic tree exhibits consistent topology, indicating that the interspecific relationships among holothuroids are monophyletic (Figure 1). Identifying the taxonomy of organisms from the perspective of mitochondrial genes avoids convergent evolution, which means that distant species can evolve similar phenotypes.

**Figure 1.**
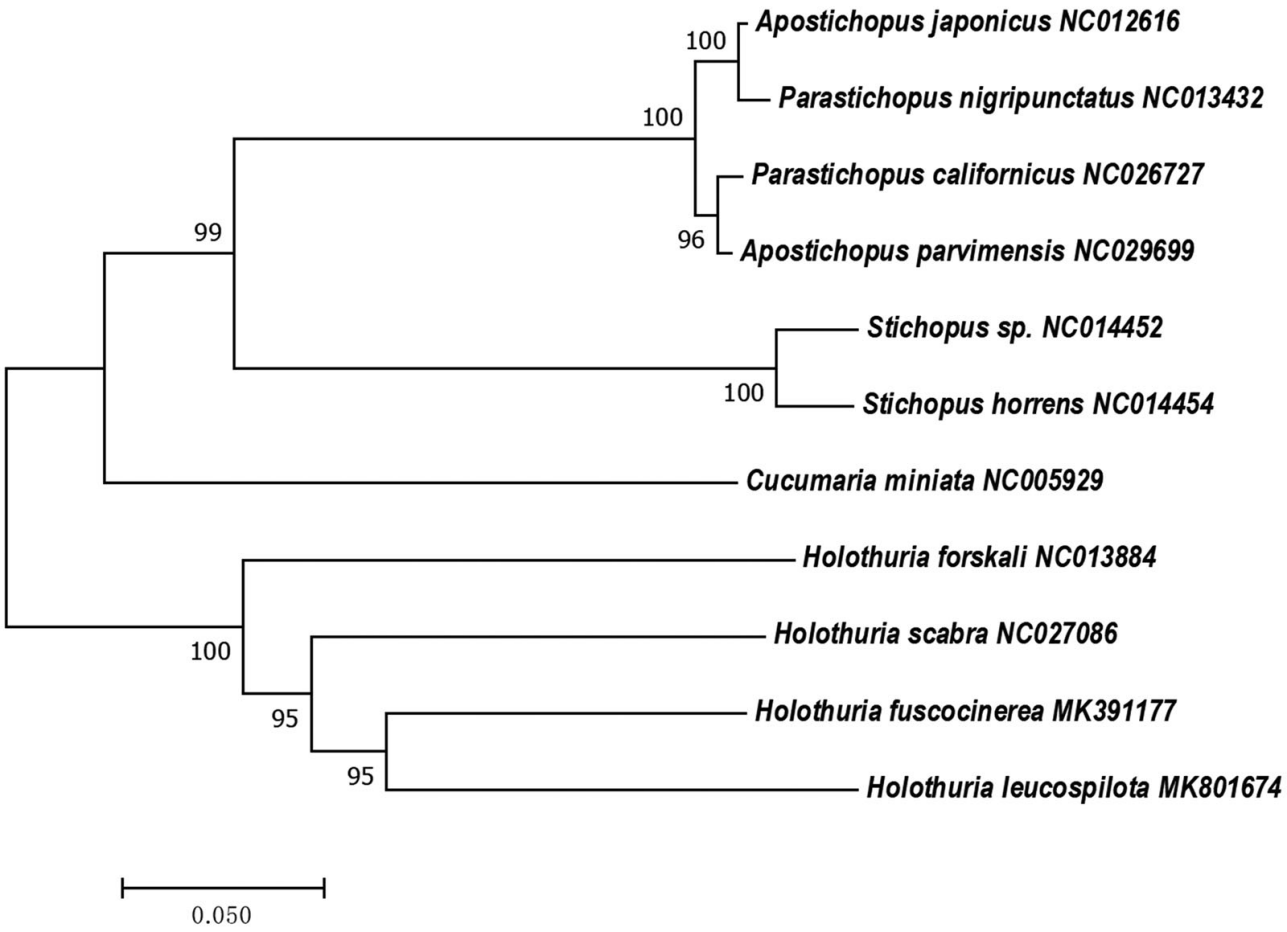
Phylogenetic tree based on the 16S rRNA in the mitochondrial genome of *Holothuria fuscocinerea*.

## Data Availability

The data that support the findings of this study are openly available in GenBank at https://www.ncbi.nlm.nih.gov/genbank/, reference number MK391177.
